# Foggy Lane Dataset Synthesized from Monocular Images for Lane Detection Algorithms

**DOI:** 10.3390/s22145210

**Published:** 2022-07-12

**Authors:** Xiangyu Nie, Zhejun Xu, Wei Zhang, Xue Dong, Ning Liu, Yuanfeng Chen

**Affiliations:** 1 China-UK Low Carbon College, Shanghai Jiao Tong University, Shanghai 201306, China; xiangyu.nie@sjtu.edu.cn (X.N.); xuzhejun@sjtu.edu.cn (Z.X.); zhangv12@sjtu.edu.cn (W.Z.); 2 Midea Group, Shanghai 201702, China; liuning22@midea.com (N.L.); chenyf94@midea.com (Y.C.)

**Keywords:** foggy scene understanding, lane detection, deep learning, synthetic dataset

## Abstract

Accurate lane detection is an essential function of dynamic traffic perception. Though deep learning (DL) based methods have been widely applied to lane detection tasks, such models rarely achieve sufficient accuracy in low-light weather conditions. To improve the model accuracy in foggy conditions, a new approach was proposed based on monocular depth prediction and an atmospheric scattering model to generate fog artificially. We applied our method to the existing CULane dataset collected in clear weather and generated 107,451 labeled foggy lane images under three different fog densities. The original and generated datasets were then used to train state-of-the-art (SOTA) lane detection networks. The experiments demonstrate that the synthetic dataset can significantly increase the lane detection accuracy of DL-based models in both artificially generated foggy lane images and real foggy scenes. Specifically, the lane detection model performance (F1-measure) was increased from 11.09 to 70.41 under the heaviest foggy conditions. Additionally, this data augmentation method was further applied to another dataset, VIL-100, to test the adaptability of this approach. Similarly, it was found that even when the camera position or level of brightness was changed from one dataset to another, the foggy data augmentation approach is still valid to improve model performance under foggy conditions without degrading accuracy on other weather conditions. Finally, this approach also sheds light on practical applications for other complex scenes such as nighttime and rainy days.

## 1. Introduction

With the rapid development of autonomous driving and assisted driving technologies, the accurate perception of dynamic traffic elements has become an essential prerequisite for reliable active safety strategies [1,2]. As a vital function of dynamic traffic perception, lane detection has gained increasing attention in recent years. Meanwhile, vision-based lane detection technology has seen significant progress, and deep neural networks, such as the mainstream technology for computer vision, have been widely utilized in lane detection [3,4,5,6,7,8,9,10]. However, the application scenarios of most deep learning (DL)-based lane detection are still limited to ideal weather conditions, e.g., clear daytime. However, little research [11,12,13,14,15,16] focuses on low-light weather conditions, such as foggy and rainy days, which are significant for increasing the adaptability of perception technology for autonomous driving vehicles. In fact, lane detection can be more challenging in complicated weather conditions. As the meteorological and lighting conditions deviate from the ideal case, both the clarity and contrast of the images decrease. Hence, problems such as color distortion and loss of fine features occur in the image, which bring about more difficulties in extracting lane features, thus affecting the accuracy of the computer vision based model [17,18]. Therefore, this paper aims to improve the accuracy of DL-based lane detection model in the complex foggy weather condition.

Over the past years, a great deal of attention has been paid to image dehazing algorithms to increase scene visibility, from traditional image processing methods [19,20,21,22,23,24,25] to convolutional neural network (CNN) based methods [26,27,28,29,30,31,32]. Though many algorithms achieve the goal of dehazing, in practice, introducing the defogging algorithm in the lane detection pipeline is computationally expensive. This means it is costly and infeasible to deploy on resource-constrained devices or in real-time applications. Hence, improving the accuracy of DL-based lane detection model without introducing an extra computation budget is crucial. Dataset synthesis is one of the most prevailing methods to help improve the performance of deep learning models. For a data-driven algorithm, the number and diversity of the training data directly influence the DL-based lane detection model’s performance. Therefore, to improve the accuracy of lane detection under complicated weather conditions, the scale of the dataset in the corresponding scenes needs to be significantly increased [33].

Several open-source annotated datasets used for traffic lane detection in autonomous driving include Caltech Lanes [34], KITTI [35], CityScapes [36], TuSimple [37], CULane [6], ApolloScape [38], BDD100K [39], LLAMAS [40], A2D2 [41], VIL-100 [42] et al. Caltech Lanes only consists of 1224 images, while the lane markings in the images of KITTI and CityScapes are not annotated; thus, these datasets are usually used for model testing and evaluation. TuSimple and LLAMAS mainly focus on highways in sunny scenes, deficient of lane marking images under complex weather conditions. Though ApolloScape and BDD100K provide large scale images under various scenarios for driving, they lack the annotation of lane markings on an instance level. It is important to label the multiple lanes in each image of these datasets as different categories in autonomous driving, especially for trajectory planning, autonomous navigation, and lane change tasks. CULane, A2D2, and VIL-100 meet the mentioned requirements and already contain several complex weather scenes. However, the foggy weather is missing in CULane and A2D2, leading to the low accuracy of the lane detection model under foggy conditions. Additionally, the scales of A2D2 and VIL-100 are relatively small, with the latter only containing 300 images in hazy weather.

**Figure 1:**
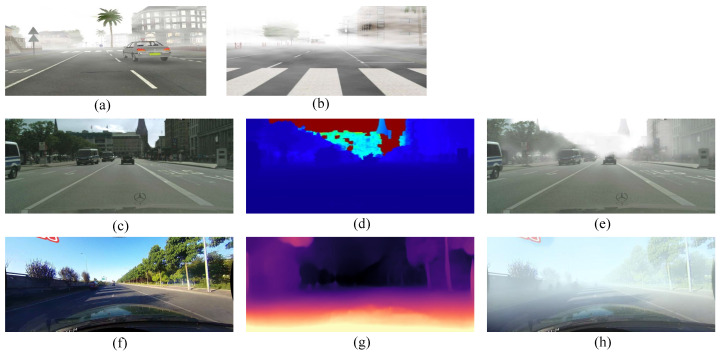
Typical samples from (**a**) FRIDA [13] and (**b**) FRIDA2 [14], (**c**) is one original clear image from Cityscapes [36], (**d**) displays its depth, and (**e**) is the simulated foggy image from Foggy Cityscapes [15]. (**f**–**h**) show the clear image, depth map, and generated foggy image from our proposed dataset, respectively.

A feasible method to enlarge the dataset under hazy weather conditions is artificially generating lane images under foggy conditions using images under clear weather. Thus, the trained model can improve its performance in all scenes. The method of augmenting existing datasets by synthesized images has been used in several scenes, such as rainy [11] and nighttime [12] conditions. Despite some previous studies in synthesizing foggy images for autonomous driving, including FRIDA [13], FRIDA2 [14], Foggy CityScapes [15], and Multifog KITTI [16], there are few studies focusing on augmenting datasets using artificially generated foggy images and its effectiveness for lane detection task. Tarel et al. constructed synthetic outdoor foggy datasets FRIDA (Foggy Road Image Database) and FRIDA2 based on numerical images using SiVIC^TM^ software. The former contains 72 foggy images with 18 original images, and the latter contains 330 images in 66 diverse scenes. However, the number of images in these two datasets is too small, and the images are also at low resolutions with poor visual effects (as shown in Figure 1), hence insufficient to support lane detection model training for higher accuracy, especially in the foggy scene. Sakaridis et al. established Foggy Cityscapes, and Mai et al. established Multifog KITTI by adding synthetic fog to images from Cityscapes and KITTI, respectively. However, the scales of these two datasets are relatively small and the annotations of the lane markings are missing. Moreover, these foggy image synthesis methods rely on image perception and depth information using complex sensors like Lidar, RGBD camera, or binocular camera, which would introduce additional costs in practice. Additionally, Figure 1d,e also shows that the quality of the generated foggy images in Foggy CityScapes suffers from incorrect depth (Figure 1d), especially in the foreground.

In this work, we propose a new dataset augmentation method, using a self-supervised monocular depth prediction method to extract the depth information from monocular lane images and then synthesize foggy images based on the atmospheric scattering model. Our proposed framework estimates the depth map only from the original image, which means the method could be easily applied to other datasets without depth information collected by extra sensors. We established the FoggyCULane dataset by enlarging the CULane dataset with the synthesis of foggy images using our method to improve the accuracy of the lane detection model in foggy scenes. Networks with outstanding lane detection performance in recent years are trained with our FoggyCULane and CULane to verify the feasibility and effectiveness of our method in different complex scenes, especially in foggy scenes. The main contributions of this paper are:

- A new dataset augmentation and synthesis method was proposed for lane detection in foggy conditions, which highly improved the accuracy of the lane detection model under foggy weather without introducing extra computational cost or complex framework for the algorithm.
- We established a new dataset, FoggyCULane, which contains 107,451 frames of labelled foggy lanes. This would help the community and researchers to develop and validate their own data-driven lane detection or dehaze algorithms.

## 2. Background

Many studies have analyzed the reasons for the loss of sharpness and contrast in foggy images through modeling. In 1924, Koschieder et al. introduced the standard optical model [43] for daytime fog, which has been used extensively in image dehazing. McCartney E J et al. [44] further reported in 1976 that the absorption and scattering of light by suspended particles (including water droplets, dust, and aerosols) in the atmosphere causes the attenuation of light transmitting between the target and the camera. Besides, the scattering of suspended particles also generates background light, which leads to a decrease in contrast and saturation of images under foggy conditions. In 1999, Nayar et al. [45] established a mathematical model to describe the atmospheric scattering process clearly. The model assumes that under a strong scattering medium (e.g., foggy scenes), firstly, the light reflected by the target is absorbed and scattered by suspended particles in the atmosphere, resulting in the attenuation of the light reflected by the target, thus decreasing the brightness and contrast of the imaging. Secondly, ambient light, such as sunlight, is scattered by the scattering medium in the atmosphere to form air light. Sometimes the intensity of this scattered light is greater than that of the target light, thus causing the image to blur.

However, the reconstruction of foggy images using the above model requires depth information from the original image. Since most current traffic lane datasets do not provide the required depth information, including the most widely used CULane dataset, the extraction of depth information needs to be performed as the first step of foggy image synthesis.

Depending on the number of viewing angles, the depth estimation of images can be divided into binocular depth estimation and monocular depth estimation. However, the binocular algorithms require multiple images of the same scene with different angles, from which the corresponding targets are matched and reconstructed in 3D.

Therefore, monocular depth prediction has been extensively studied in the field of computer vision in recent years. Based on the basic assumption that there are mapping relationships between RGB images and depth maps, data-driven deep learning methods have been proposed and applied to monocular depth estimation problems. Among them, deep learning techniques represented by Convolution Neural Network (CNN) have made substantial progress and gradually become the mainstream method for monocular image depth estimation [46,47,48,49,50].

Supervised by images with depth annotations, Eigen et al. [51] first trained a CNN-based depth estimation model and realized monocular depth estimation using the deep learning method. They proposed to estimate the depth of a monocular image using two scales of neural networks: a coarse-scale network to predict the global depth of the image and a fine-scale network to optimize the local details.

In fact, acquiring large amounts of diverse images with exact depth information is challenging, and supervised learning cannot adapt to changing environments. Supervised learning methods require that each RGB image has its corresponding depth label, and the depth label annotation usually requires depth cameras or light detection and ranging (LIDAR), which are limited by detection range and cost, respectively. In addition, depth labels acquired from LIDAR are usually sparse points that are difficult to match with the original pixel-level image. Therefore, unsupervised or self-supervised depth estimation methods that do not require depth labels have been a research trend in recent years. One of the most prominent methods is the Monodepth2 network proposed by Godard et al. [52].

## 3. Methods

**Figure 2:**
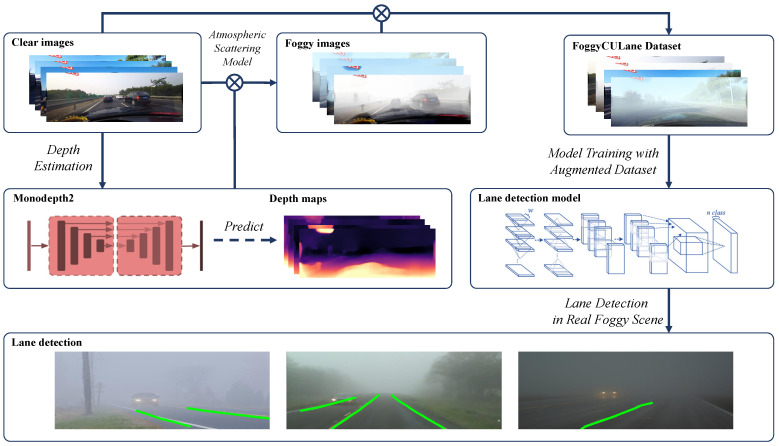
The pipeline to improve the performance of lane detection model in foggy scene with our method.

In this paper, we proposed a dataset enhancement method for lane detection in foggy conditions based on the original CULane dataset, using a self-supervised monocular depth prediction method to extract the depth information from monocular lane images and synthesize foggy images based on the atmospheric scattering model. The main framework of our method is shown in Figure 2.

### 3.1. The Standard Optical Model of Foggy Images

From Koschmieder’s law [43], the light intensity of the target observed by the camera mainly contains two parts: the light reflected by the object that reaches the detection system after attenuation by the transmission medium and the background light formed by particle scattering. The model of imaging in foggy (atmospheric scattering) conditions is defined by Koschmieder’s law as follows:

(1) I(x)=J(x)t(x)+A(1−t(x))

where x indicates one certain pixel of the image, I(x) refers to the observed foggy image of the object at the pixel x, and J(x) refers to the original clear image of the object. A is the global skylight representing the ambient light in the atmosphere and is generally assumed to be a constant. Additionally, t(x) determines the transmission of the light from the object in the atmosphere. Assuming the medium is homogeneous, the transmission t(x) can be further determined as:

(2) t(x)=exp(−βl(x))

where l(x) is the distance between the object and observer, β is the extinction coefficient, and larger β represents the thicker fog.

It can be seen that image degradation and information loss increase with depth l(x). Therefore, to generate synthetic fog images from the original clear scene, the key is to obtain the value of l(x) in (2), that is, the depth information of the clear images needs to be extracted.

### 3.2. Monocular Depth Estimation

With the rapid development in deep learning, deep neural networks have shown excellent performance in recovering the pixel-level depth map from a single image [46,47,48,49,50]. In this paper, the Monodepth2 network (trained on the KITTI dataset) proposed by Godard et al. [52] is used for depth extraction of clear images in the CULane dataset, which is, in turn, employed to generate foggy images.

Monodepth2 is a self-supervised monocular depth estimation network that combines depth estimation and pose estimation to achieve pixel-level SOTA depth predictions. The recovery of the depth information was achieved based on the photo-consistency assumption, i.e., points in the same space should also have the same luminosity in the projection of different views. Based on minimum reprojection loss, the full-resolution multi-scale sampling method, and auto-masking loss, Monodepth2 can achieve outstanding depth estimation while trained with either monocular or binocular images as input data.

The loss functions of Monodepth2 are defined as follows:

(3) $$\left\{ \begin{array}{l} I_{t^{\prime }\to t}=I_{t^{\prime }}\langle \;proj(D_{t},T_{t\to t^{\prime }},K)\rangle \\ pe\left(I_{a},I_{b}\right)=\frac{\alpha }{2}\left(1-\mathrm{SSIM}\left(I_{a},I_{b}\right)\right)+\left(1-\alpha \right)∥I_{a}-I_{b}∥\\ L_{p}=\underset{t^{\prime }}{min}\;pe\left(I_{t},I_{t^{\prime }\to t}\right)\\ L_{s}=\left|\partial_{x}d_{t}^{*}\right|e^{-\left|\partial_{x}I_{t}\right|}+\left|\partial_{y}d_{t}^{*}\right|e^{-\left|\partial_{y}I_{t}\right|},d_{t}^{*}=d_{t}/\bar{d_{t}}\\ L=\mu L_{p}+\lambda L_{s} \end{array}\right.$$

where $I_{t^{\prime }\to t}$ is defined as the relative pose for each source view $I_{t^{\prime }}$ with respect to the target image $I_{t}$’s pose; proj( ) projects the predicted depth map $D_{t}$ in resulting 2D coordinates with $I_{t}^{’}$ and 〈 〉 is the sampling operator; K represents pre-computed intrinsics for simplicity; pe is the photometric error function constructed by L1-norm and Structural Similarity(SSIM); $d_{t}^{*}$ is the mean-normalized inverse depth; $L_{s}$ is edge-aware smoothness and $L_{p}$ is the per-pixel photometric loss; μ and λ are coefficients corresponding to auto-masking stationary pixels and multi-scale estimation respectively; L is the final loss function.

Considering that our purpose is to generate foggy images whose density varies with distance, it is unnecessary to obtain the real and absolute depth information. Instead, the trend of depth variation or the relative variation of depth meets the requirement. Therefore, Monodepth2 is sufficient for this current study.

### 3.3. Foggy Image Generation

To adapt to the relative depth, the actual transmission value t’(x) is reformed as:

(4) $$t^{\prime }\left(x\right)=1-\exp\left(-\beta \left(1-l^{\prime }\left(x\right)\right)\right)$$

**Figure 3:**
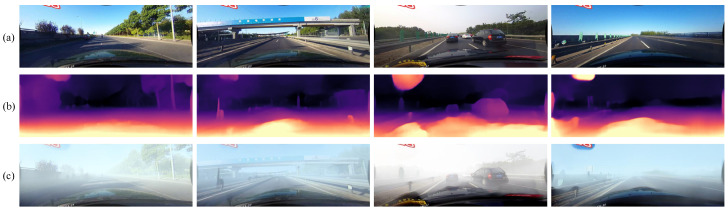
Examples of depth estimation and foggy image synthesis, (**a**) original images from CULane, (**b**) depth maps predicted by Monodepth2, and (**c**) synthetic foggy images.

Here, $l^{\prime }\left(x\right)$ is the depth matrix in the range [0, 1], which indicates the normalized depth estimated by Monodepth2. Substituting (2) with (4) we can acquire the observed foggy image of the object *I(x)*. The synthetic monocular depth map and foggy images acquired using the above method are shown in Figure 3. The acquired depth map can clearly represent the depth of each part of the original image and the trend of depth change. The generated foggy images look like real foggy pictures from the driver’s perspective.

### 3.4. Establishment of FoggyCULane Dataset

**Figure 4:**
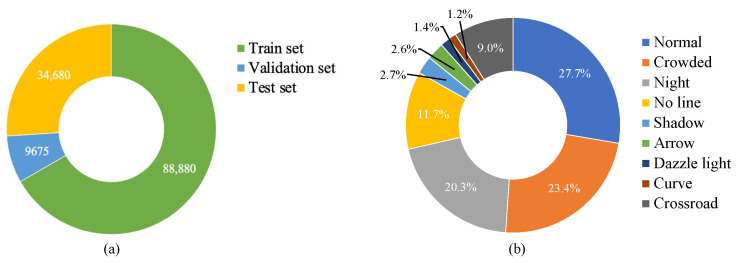
The overall structure of CULane dataset, (**a**) the number of images in each set, (**b**) proportion of each scene.

Figure 4 presents the overall structure of the original CULane dataset, which involves 133,235 pictures of traffic lanes collected by cameras mounted on different vehicles, among which 88,880 for the training set, 9675 for the validation set, and 34,680 for the test set. The test set is divided into the normal scene and eight challenging categories, including crowded, night, no line, shadow, arrow, dazzle light, curve, and crossroad.

The CULane dataset focuses on the current lane and up to 4 adjacent lane markings, which are the most concerned lanes during driving. The traffic lanes are manually annotated with cubic splines for each image, and the labels are recorded as a set of coordinate points. The lanes are still annotated based on semantic information for cases where the lane markings are obscured or not visible by vehicles.

**Figure 5:**
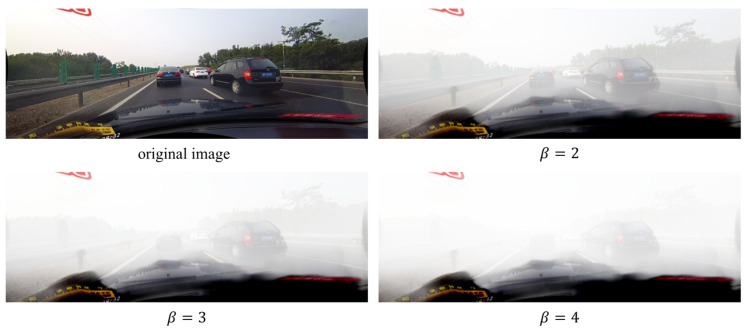
Example of synthetic foggy images under three different densities (β=2, 3, 4), together with their clear image, with the thickness of fog increasing with β.

Through the foggy image generation method, we expand the training set, validation set, and test set of CULane, respectively. Considering the diversity and complexity of fog density in the real foggy scene, we attempt to simulate fog scenes more realistically and adapt the dataset to multiple density scenes. Through experimentation, we found that the values of β should be limited to a reasonable range, or the artificially generated fog would be almost invisible or too thick. Thus, β is finally set to 2, 3, and 4, respectively, to generate three levels of foggy scenes with increasing fog density, as shown in Figure 5. Meanwhile, because we use the original images in the CULane dataset, the lane annotations are still applicable to the synthetic images.

We selected 16,532 images from the CULane train set as the original inputs for foggy image generation and processed them separately with different fog densities (β = 2, 3, 4). Together with the original lane images, the total number of the training set for our FoggyCULane was 138,476. Similarly, while keeping the original test set images, we selected all the images in the normal scene to synthesize the foggy images and form the new test set with 63,510 images. As for the validation set, all the 9675 images were used for foggy image generation, resulting in 38,700 images in the new validation set. Table 1 presents the file structure of our synthesized foggy images in FoggyCULane based on the original CULane dataset, while Table 2 shows the comparison between test sets in CULane and FoggyCULane.

## 4. Experiments

### 4.1. Dataset

There were 186,786 images in total in our FoggyCULane dataset. To verify the effectiveness of the synthetic dataset in improving the performance of lane detection networks, we used the Spatial CNN (SCNN) network as the baseline method to investigate the datasets with different densities of fog. Other state-of-art methods were also employed to evaluate the improvement of the FoggyCULane dataset. Additionally, 300 hazy images with annotated lane makings in the VIL-100 dataset were employed to evaluate the effectiveness of our proposed framework. Despite of the hazy images in VIL-100, we also collected 182 traffic lane images under real thick foggy weather to test whether the trained SCNN network is adequate for more challenging real foggy scenes. Moreover, the fog synthetization method was also applied to another dataset, i.e., VIL-100, to test its adaptability.

### 4.2. Evaluation Metrics

To quantitatively evaluate the lane detection results, we refer to the methods in [6], where both the real lane annotation and the predicted lane marking are considered a line area with a width of 30 pixels, and the Intersection over Union (IoU) between them is calculated. In this paper, the IoU threshold is set to 0.5. When the IoU between the predicted lane marking and the real lane is not less than 0.5, the prediction is considered as True Positive (TP), and the opposite is considered as False Positive (FP); accordingly, True Negative (TN) and False Negative (FP) can also be defined.

As mentioned above, the expanded test set of FoggyCULane had 12 different complex scenes, including the newly added three different density foggy scenes. Here, the F1-measure is employed to be the index for quantitative evaluation and is defined as follows:

(5) $$F1\;\mathrm{score}=2\times \frac{\mathrm{Precision}\times \mathrm{Recall}}{\mathrm{Precision}+\mathrm{Recall}},\;\begin{array}{c} \mathrm{Precision}=\frac{TP}{TP+FP}\\ Recall=\frac{TP}{TP+FN} \end{array}$$

### 4.3. Implementation Details

SCNN is a spatial convolutional neural network creatively proposed by Xingang Pan et al. [6], which is reported to have the highest lane detection accuracy in the CULane dataset. Here, the model was trained with Stochastic Gradient Descent (SGD) as the optimizer with a base learning rate of 0.01 and momentum of 0.9. Weight decay is set to be $10^{-4}$. The training and testing were undertaken on 8 NVIDIA GTX 2080Ti GPUs and an Intel Xeon E5-2682 v4 CPU. Before training, the images were resized to 800 × 288 for the limited memory of the GPU. All the models were downloaded from public source code with default hyper-parameters and pre-trained as previous works did.

### 4.4. Effect of FoggyCULane Dataset on SCNN

Table 3 shows the results of five epochs of training of the SCNN network on different datasets, including 12 different scenes. The last three rows are the newly added three foggy scenes with increasing fog densities. The data in each column is acquired from five epochs of neural network trained with different datasets, including the original CULane dataset, the FoggyCULane dataset with three foggy densities of values 2, 3, and 4, and the FoggyCULane dataset with a mixture of the three foggy densities. The values in the table (except the Crossroad row) represent the F1-measure values in percentage for each scene, while the Crossroad row shows FP values.

**Figure 6:**
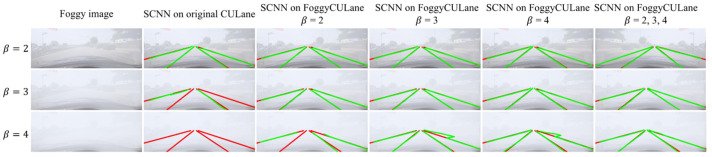
The foggy lane detection results of SCNN trained on different datasets show the real lane markings in red and the predicted lane markings in green.

As shown in Table 3, the models trained with the FoggyCULane dataset practically obtained better lane detection performance than those trained with the original CULane dataset under the three foggy conditions, especially with the highest density (β = 4). Figure 6 presents the one of the lane detection results of SCNN trained on different datasets.

Under the heaviest foggy weather condition, the lane detection performance (F1-measure) increased from 11.09 to 89.21 by FoggyCULane. Particularly, the model trained by the FoggyCULane dataset with the mixing of the three densities of foggy weather images achieved the best performance (86.65, 81.53, and 70.41 in F1-measure) in all foggy scenes. This improvement is due to the addition of foggy images to the training set, allowing the neural network to extract features of lane markings under foggy weather for learning. These synthesis haze images contain enough foggy information to satisfy the need for deep learning. Therefore, this study can significantly improve the lane detection performance of neural networks in foggy scenes while not negatively affecting lane detection in other complex scenes. By comparing three different density FoggyCULane datasets (β = 2, 3, 4), it can be observed that:

- The dataset with mixed foggy densities has better performance than the dataset with a single foggy density. On the one hand, there are more haze images in the dataset with mixed fog densities, and the neural network can be more exposed to the foggy scene when training. Therefore, it is more sensitive to lane markings under foggy weather. On the other hand, the dataset with mixed fog densities contains fog images of three densities, making the network learn and extract features for lane detection in foggy condition comprehensively during model training.
- The model trained in the dataset with the corresponding fog density value achieves the best lane detection performance in each foggy scene. This indicates that the features extracted by the network vary with fog densities. Therefore, the dataset with mixed fog densities should be applied to obtain a better performance in practice.

### 4.5. Effect of FoggyCULane Dataset on Other State-of-Art Methods

To verify the effectiveness of the FoggyCULane dataset on other detection network, four other state-of-art lane detection networks (ENet-SAD [7], ERFNet [8], LaneATT [9], and LaneNet [10]) were also trained with the original CULane dataset and FoggyCULane dataset (including all the densities of fog) respectively, then tested under normal and foggy scenes to compare their performance.

As shown in Table 4, all the models trained by the FoggyCULane dataset have improved performance under foggy conditions compared with the models trained on the original CULane. The test results of these networks are similar to the results of the SCNN network, which also demonstrates the generality and applicability of our method for different lane detection networks.

### 4.6. Ablation Study

#### 4.6.1. Lane Detection Results in Real Foggy Scene

**Figure 7:**
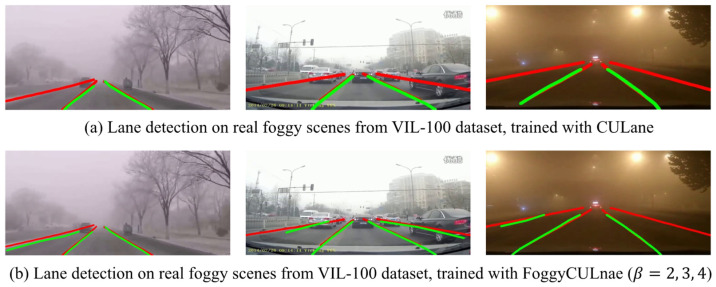
The lane detection results of SCNN tested in real foggy scenes from VIL-100 dataset. Red lines denote the real lane markings and green lines denote the predicted lane markings.

VIL-100 dataset was collected for lane detection by Zhang et al. [42], which includes 10,000 frames in different real traffic scenarios including normal, crowded, curved road, damaged road, shadows, road markings, dazzle light, haze, night and crossroad, and multiple scenarios may simultaneously occur in a single frame. The VIL-100 dataset contains three hazy scenes (here named as real foggy scene 1, real foggy scene 2, and real foggy scene 3) with 100 images in each scene, and the representative images in each scene are shown in Figure 7. To further evaluate the effectiveness of our proposed framework in improving the performance of the lane detection model in real foggy scenes, 300 real foggy images with annotated lane markings from the VIL-100 dataset were adopted. SCNN model trained on CULane and FoggyCULane (β=2, 3, 4) were tested on these foggy images, respectively. Figure 7 also compares the lane detection results from the two models, and Table 5 presents the values of F1-mearsure to evaluate the performance of the models in these foggy images. As shown in Table 5, the performance of SCNN trained on FoggyCUlane was generally improved in three scenes, especially in foggy scene 1 and foggy scene 3, in which the fog is thicker, and the fog is further overlaid with night darkness. The result further indicates that our proposed framework could enhance the ability of lane detection models in real foggy scenes.

Since the fog in the selected images of VIL-100 are commonly thin, to further evaluate whether the trained lane detection model is adequate for real foggy scenes, 182 lane line images containing more challenging real foggy scenes were downloaded and compiled from the Internet. We used the SCNN models trained on the original CULane dataset and the FoggyCULane (β=2, 3, 4) dataset to verify the model’s performance on real-world foggy images. These challenging real foggy images have several characteristics:

1. The fog density in the image varies among images, and the fog density is also not uniform in the same image.
2. The angle and orientation of the images vary greatly from one another, including images taken from the perspective of roadside pedestrians, road surveillance cameras, and in-vehicle recorders.
3. Vehicles and pedestrians in the images occlude the lane marks to varying degrees, and the number of lane marks in each image is not the same.

Since these real fog lane images collected from the Internet do not have lane labels, the same strategy as the Crossroad scene in the CULane test set was used here to evaluate the lane detection results using FP values.

Comparing the FP values of the above two models, we found that the performance of the SCNN model trained with the FoggyCULane dataset is significantly better than the other on real foggy days. The FP value increases from 33 to 118, as shown in Table 6, meaning the percentage of images with recognized lane markings increased from 18.13% to 64.84%.

**Figure 8:**
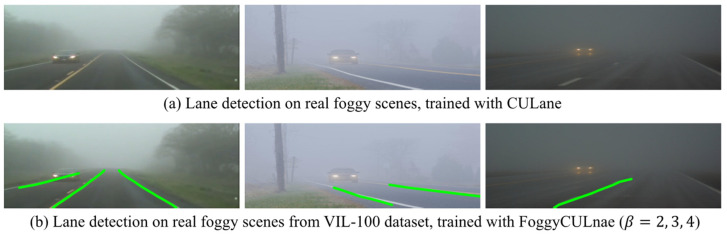
The lane detection results of SCNN tested in real foggy images acuiqred online without labelling: the first row shows the results of SCNN trained on CULane and the second row shows the results of SCNN trained on FoggyCULane. Green lines denote detected lane markings by the model.

Several representative lane detection results in foggy weather are shown in Figure 8. It can be seen from Figure 8a that for multiple and obstructed lane markings, the model still detects the lane marking blocked by the vehicle based on semantic information. Figure 8b shows the image taken from the perspective of the pedestrian on the roadside. Unlike other images, the lane marking is not located in the central area of the image, with a different angle and direction. Figure 8 shows the case of low light and high fog density, yet still, one of the lanes is detected in this complex scenario. Overall, the SCNN model trained with the FoggyCULane dataset with three different densities of foggy scenes can achieve lane detection for more complex, realistic foggy scenes.

#### 4.6.2. Application of Proposed Framework on VIL-100 Dataset

**Figure 9:**
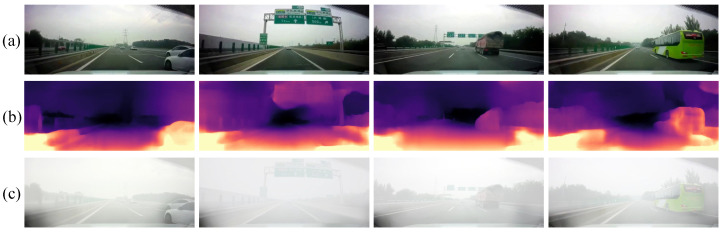
Examples of depth estimation and generated foggy images (β=3) from VIL-100, (**a**) original images, (**b**) predicted depth maps, and (**c**) synthesized foggy images.

In this section, we applied our data augmentation framework to the VIL-100 dataset and generated FoggyVIL-100 to evaluate the feasibility of the current method further. We synthesized 5400 foggy images from the normal scenes in the VIL-100 dataset. The generated images contained three levels of simulated fog (β=2, 3, 4), with 1800 images at each fog intensity level. Figure 9 shows the results of our generated foggy images on VIL-100 and the corresponding clear images and depth maps. The generated foggy images of all three foggy densities were mixed with the original images to establish a new dataset named FoggyVIL-100 to further test the effectiveness and feasibility of our proposed framework. Table 7 presents the file structure of VIL-100 and FoggyVIL-100, while Table 8 presents the comparison of scenarios in test sets in VIL-100 and FoggyVIL-100. Note that 300 hazy images in the original VIL-100 (real foggy scene 1, real foggy scene 2, and real foggy scene 3) were picked out to evaluate the performance of the lane detection model trained on different datasets.

The SCNN model was trained on the original VIL-100 dataset and FoggyVIL-100 dataset with default hyperparameters, respectively. Table 9 shows the lane detection results (F1-measure) of the SCNN model on the FoggyVIL-100 test set. As shown in Table 9, compared with the SCNN trained on original VIL-100, that trained on FoggyVIL-100 improved F1-measure in all scenes, especially in generated foggy scenes, which were improved from 72.84 to 81.57, 54.00 to 79.22, and 13.57 to 66.19 under three fog densities, respectively. Meanwhile, the model performance in other weather conditions was not degraded. In fact, it mostly improved, which may be the result of the increased scale and enriched features of the trained set, especially for crowed, dazzl light, and night scenes, in which the light conditions are commonly low.

Table 9 shows that the performance of the SCNN model trained on FoggyCULane was improved in the FoggyVIL-100 test set. This suggests that even when the camera position or level of brightness was changed from one dataset to another, the foggy data augmentation approach is still valid to improve model performance.

Moreover, the models trained on VIL-100 and FoggyVIL-100 were also evaluated in the FoggyCULane test set, with the results depicted in Table 10. Table 10 also shows that the performance of the model was improved by training on the augmented dataset, especially for the heaviest simulated foggy scene (from 11.10 to 36.51). Table 11 and Table 12 present the evaluation results in real foggy scenes collected from the original VIL-100dataset and the internet, respectively. As is shown in Table 11, the augmented dataset improved the performance of the SCNN model in three real foggy scenes by 12.41%, 3.35%, and 15.07%, respectively, and the FP values in real foggy images collected from the internet were also improved from 24 to 77 as shown in Table 12. The results in this section further proved the feasibility and effectiveness of our method. 

## 5. Conclusions

In this paper, we focus on improving the lane detection performance of neural networks in foggy conditions. We proposed a data augmentation method to synthesize foggy images from the clear weather condition. A total of 107,451 foggy images are synthesized based on the CULane dataset using our method; thus, the scale of the original CULane dataset is expanded 1.8 times to solve the problem of insufficient lane dataset under foggy weather. Therefore, a new lane detection dataset, FoggyCULane containing three different densities of foggy weather scenarios, was built.

The results of our work indicate that artificially synthesizing images to expand the dataset can significantly improve the performance of lane detection in the corresponding complex scene. Accuracy in the F1-measure of the SCNN model in different densities of foggy weather was improved from 74.65, 51.41, and 11.09 to 86.65, 81.53, and 70.41, respectively. The improvement is especially notable in the higher fog density condition; meanwhile, the expansion of the dataset does not negatively affect the performance of other complex scenes. Aside from the SCNN model, we also evaluated our method on other SOTA networks. All the models trained on the FoggyCULane dataset had improved performance under foggy conditions, demonstrating the generality and applicability of the method.

The performance of our method under real foggy scenes also showed significant improvement, with the F1-measure increasing from 59.13, 57.01, and 40.15 to 66.82, 59.44, and 46.08 in three real foggy scenes, respectively. Besides, this data augmentation method was further applied to another dataset, VIL-100, to test the adaptability of this approach. Similarly, it was found that even when the camera position or level of brightness was changed from one dataset to another, the foggy data augmentation approach is still valid for improving model performance under foggy conditions without degrading the accuracy on other weather conditions. Therefore, the method proposed in this paper can effectively improve the lane detection performance in foggy scenes for both highways and more complex urban roads without introducing extra computation on resource-constrained devices.

In summary, a data augmentation method is proposed in this work to improve the performance of DL-based lane detection algorithms under foggy weather. This work is of great practical significance for the following reasons. Firstly, the foggy image synthesis in our method avoids introducing extra sensors to acquire depth information, which means the method could be easily deployed in vision-based lane detection datasets. Additionally, despite the fact that this work mainly focuses on the lane detection task under foggy weather, it is expected that the proposed method could be further extended to improve object recognition and semantic segmentation tasks under other complex scenes for future study.

## Figures and Tables

**Table 1:** The overall file structure of the Foggy CULane dataset compared with the original CULane dataset, β is set to 2, 3, and 4 for simulated fog, and β=1 represents the original clear image.

	β	Train Set Images	Validation Set Images	Test Set Images	Total Images
CULane	1	88,880	9675	34,680	133,235
Foggy CULane	1	88,880	9675	34,680	133,235
	2	16,532	9675	9610	35,817
	3	16,532	9675	9610	35,817
	4	16,532	9675	9610	35,817

**Table 2:** Comparison between test sets of CULane and FoggyCULane  (β=2, 3, 4).

Scenarios	CULane Test Set	FoggyCULane Test Set
Normal	9610	9610
Crowded	8115	8115
Night	7040	7040
No line	4058	4058
Shadow	936	936
Arrow	900	900
Dazzle light	486	486
Curve	415	415
Crossroad	3120	3120
Foggy_beta2	0	9610
Foggy_beta3	0	9610
Foggy_beta4	0	9610

**Table 3:** Evaluation results (F1-measure) of SCNN tested in different scenes, with the model trained with different datasets.

Category	SCNN Trained on Origin CULane	SCNN Trained on FoggyCULane with β = 2	SCNN Trained on FoggyCULane with β = 3	SCNN Trained on FoggyCULane with β = 4	SCNN Trained on FoggyCULane with β = 2, 3, 4
Normal	89.21	89.82	89.81	89.20	90.09
Crowded	68.11	68.30	68.63	67.93	69.53
Night	64.85	65.49	64.00	64.24	65.08
No line	43.25	43.21	43.22	43.02	42.80
Shadow	59.27	58.31	61.40	56.57	63.77
Arrow	83.29	84.51	84.30	83.82	83.66
Dazzle light	59.93	65.64	64.07	58.74	61.16
Curve	62.65	65.61	66.51	62.51	62.09
Crossroad (FP)	2704	2679	3155	2386	3004
Foggy_beta2	74.65	86.63	85.58	83.74	86.65
Foggy_beta3	51.41	79.53	80.07	77.56	81.53
Foggy_beta4	11.09	60.33	65.27	65.91	70.41

**Table 4:** Evaluation results (F1-measure) of other lane detection models, trained with CULane and FoggyCULane (*β* = 2, 3, 4), respectively.

Category	ENet-SAD		ERFNet		LaneATT		LaneNet	
	CULane	Foggy CULane	CULane	Foggy CULane	CULane	Foggy CULane	CULane	Foggy CULane
Normal	88.52	89.64	87.46	88.57	85.06	87.03	88.13	88.95
Foggy_beta2	71.86	85.21	69.56	84.34	69.20	84.99	65.24	83.51
Foggy_beta3	50.62	80.35	49.73	79.42	47.14	78.21	41.47	77.32
Foggy_beta4	12.13	71.32	10.23	65.28	10.93	66.45	9.82	59.48

**Table 5:** Lane detection results (F1-measure averaged from 100 images) of SCNN on real foggy scenes from VIL-100 dataset, with the models trained on CULane and FoggyCULane, respectively.

	SCNN Trained on CULane	SCNN Trained on FoggyCULane
Real foggy scene 1	59.13	66.82
Real foggy scene 2	57.01	59.44
Real foggy scene 3	40.15	46.08

**Table 6:** Lane detection results (FP) of SCNN in real foggy scene.

	SCNN Trained on CULane	SCNN Trained on FoggyCULane
FP	33	118

**Table 7:** The overall file structure of the FoggyVIL-100 dataset compared with the original VIL-100 dataset, β is set to 2, 3, and 4 for simulated fog, and β=1 represents the original clear image.

	β	Train Set Images	Test Set Images	Total Images
VIL-100	β=1	8000	2000	10,000
Foggy VIL-100	β=1	8000	2000	10,000
	β=2	1400	400	1800
	β=3	1400	400	1800
	β=4	1400	400	1800

**Table 8:** Comparison between test sets of VIL-100 and FoggyVIL-100 (β=2, 3, 4).

Scenarios	VIL-100 Test Set	FoggyVIL-100 Test Set
Normal	400	400
Crowded	700	700
Curved road	700	700
Damaged road	100	100
Shadows	200	200
Road markings	400	400
Dazzle light	200	200
Night	100	100
Crossroad	100	100
Foggy_beta2	0	400
Foggy_beta3	0	400
Foggy_beta4	0	400

**Table 9:** Lane detection results (F1-measure) of SCNN tested on different scenes of the FoggyVIL-100 test set, with the model trained with different datasets.

Category	SCNN Trained on Origin VIL-100	SCNN Trained on FoggyVIL-100 Beta = 2, 3, 4	SCNN Trained on Origin CULane	SCNN Trained on FoggyCULane Beta = 2, 3, 4
Normal	84.31	87.41	78.94	82.03
Crowded	72.63	74.47	59.89	61.22
Curved road	65.09	65.13	61.39	62.03
Damaged road	40.63	41.86	41.98	42.21
Shadows	43.05	52.35	49.56	54.32
Road markings	76.21	77.48	71.92	72.01
Dazzle light	55.05	56.31	56.25	57.64
Night	56.45	56.70	58.71	58.90
Crossroad	60.68	60.41	60.29	63.76
Foggy_beta2	72.84	81.57	62.81	73.25
Foggy_beta3	54.00	79.22	50.03	69.89
Foggy_beta4	13.57	66.19	10.42	55.14

**Table 10:** Lane detection results (F1-measure) of SCNN tested on simulated foggy scenes from FoggyCULane dataset, with the model trained on VIL-100 dataset.

Category	SCNN Trained on Origin VIL-100	SCNN Trained on FoggyVIL-100 Beta = 2, 3, 4
Foggy_beta2	52.35	57.63
Foggy_beta3	48.58	51.26
Foggy_beta4	11.10	36.51

**Table 11:** Lane detection results (F1-measure averaged from 100 images) of SCNN on real foggy scenes from VIL-100 dataset, with the models trained on VIL-100 and FoggyVIL-100, respectively.

	SCNN Trained on VIL-100	SCNN Trained on FoggyVIL-100
Real foggy scene 1	60.02	67.45
Real foggy scene 2	53.81	55.68
Real foggy scene 3	38.74	44.59

**Table 12:** Lane detection results of SCNN on real foggy scenes collected from internet.

	SCNN Trained on VIL-100	SCNN Trained on FoggyVIL-100
FP	24	77

## Data Availability

Data available on request from the authors.
